# Summarising and validating test accuracy results across multiple studies for use in clinical practice

**DOI:** 10.1002/sim.6471

**Published:** 2015-03-20

**Authors:** Richard D. Riley, Ikhlaaq Ahmed, Thomas P. A. Debray, Brian H. Willis, J. Pieter Noordzij, Julian P.T. Higgins, Jonathan J. Deeks

**Affiliations:** ^1^ Research Institute of Primary Care and Health SciencesKeele UniversityStaffordshireST5 5BGU.K.; ^2^ MRC Hub for Trials Methodology Research, School of Health and Population SciencesUniversity of BirminghamBirminghamU.K.; ^3^ Julius Center for Health Sciences and Primary CareUniversity Medical Center UtrechtUtrechtThe Netherlands; ^4^ School of Health and Population SciencesUniversity of BirminghamBirminghamU.K.; ^5^ Department of Otolaryngology – Head & Neck Surgery, Boston Medical CenterBoston University – School of MedicineBostonMAU.S.A.; ^6^ School of Social and Community MedicineUniversity of BristolBristolU.K.

**Keywords:** meta‐analysis, test accuracy, prognostic, diagnostic, calibration, discrimination

## Abstract

Following a meta‐analysis of test accuracy studies, the translation of summary results into clinical practice is potentially problematic. The sensitivity, specificity and positive (PPV) and negative (NPV) predictive values of a test may differ substantially from the average meta‐analysis findings, because of heterogeneity. Clinicians thus need more guidance: given the meta‐analysis, is a test likely to be useful in new populations, and if so, how should test results inform the probability of existing disease (for a diagnostic test) or future adverse outcome (for a prognostic test)? We propose ways to address this. Firstly, following a meta‐analysis, we suggest deriving prediction intervals and probability statements about the potential accuracy of a test in a new population. Secondly, we suggest strategies on how clinicians should derive post‐test probabilities (PPV and NPV) in a new population based on existing meta‐analysis results and propose a cross‐validation approach for examining and comparing their calibration performance. Application is made to two clinical examples. In the first example, the joint probability that *both* sensitivity and specificity will be >80% in a new population is just 0.19, because of a low sensitivity. However, the summary PPV of 0.97 is high and calibrates well in new populations, with a probability of 0.78 that the true PPV will be at least 0.95. In the second example, post‐test probabilities calibrate better when tailored to the prevalence in the new population, with cross‐validation revealing a probability of 0.97 that the observed NPV will be within 10% of the predicted NPV. © 2015 The Authors. *Statistics in Medicine* Published by John Wiley & Sons Ltd.

## 1 Introduction

Test accuracy studies aim to evaluate the performance of a candidate medical test for either diagnosing the presence of a particular clinical condition (‘diagnostic test’) or identifying those likely to experience a particular outcome in the future (‘prognostic test’). Such tests include measurable variables such as biomarkers, blood pressure and temperature, or may reflect a clinician's judgement after a physical examination or imaging result. When multiple studies evaluate the performance of a potential diagnostic or prognostic test, meta‐analysis methods can synthesise the study results to help establish if and how the test can be used in practice 1, 2, 3, 4. Current meta‐analyses of test accuracy studies focus on producing pooled estimates that summarise *average* test performance across the multiple studies 2, 3, 4, 5, 6. This typically leads to a single pooled estimate for each of sensitivity, specificity, positive predictive value (PPV) and negative predictive value (NPV) and sometimes a summary ROC curve. For example, in patients undergoing a thyroidectomy, Noordzij *et al.* 7 examine the accuracy of the % change in parathyroid (PTH) levels (from pre‐surgery to post‐surgery) for identifying those at high risk of becoming hypocalcemic within 48 h. They synthesise data from six studies and summarise that a 70% or greater reduction in PTH has a sensitivity of 0.93, a specificity of 0.88, a PPV of 0.74 and an NPV of 0.97. However, when data are available from multiple studies, a major concern is heterogeneity in test accuracy, which refers to genuine differences in the accuracy of a test from study to study. Heterogeneity in test accuracy is common in meta‐analysis and is caused by study differences in, for example, clinical settings (such as hospitals), patient selection (such as symptom severity and previous testing), choice of healthcare practitioners implementing/reading the test, the included patient populations (e.g. with a different underlying prevalence of disease) and the test methodology employed (e.g. choice of threshold, method of measurement and the reference standard).

Where heterogeneity exists, this is usually accounted for by using random‐effects models that allow test accuracy to vary across studies 2, 4. However, typically, the aim of meta‐analysis is as before: to estimate the *average* test performance across all the populations included. Although this is a worthwhile research question, the translation of such *average* meta‐analysis results into clinical practice is potentially problematic. For example, a diagnostic test may have high sensitivity and specificity values on average, but the causes of heterogeneity may lead to substantially lower values in some particular populations, leading to poor performance. Similarly, average post‐test probabilities from a prognostic test may be inaccurate, for example, if the outcome prevalence in a particular population is markedly different than the average outcome prevalence across all populations. For example, Leeflang *et al.* 5 said ‘when a review contains studies with a broad range of prevalence, there will be difficulties in working out how the average predictive values (estimated for the average included study with an average prevalence) can be applied in clinical practice.’

To address this concern, this article suggests ways to improve the translation of meta‐analysis results for clinical practice, by going beyond presenting just average test accuracy results. There are two main objectives. Firstly, we emphasise the importance of deriving and reporting prediction intervals for the potential accuracy of a test in a new population 8, 9. Secondly, we propose a cross‐validation approach for examining how to derive PPV and NPV in new populations based on existing meta‐analysis results. Our viewpoint is similar to Willis and Hyde 10, who propose that meta‐analysis results need to be tailored before use in a particular population, and Leeflang *et al.* 5, who propose predicting PPV and NPV in new populations. Our cross‐validation framework provides a way to examine the calibration of approaches (or statistical equations) that tailor (or predict) results for new populations.

The remainder of the paper is as follows. In Section 2, we introduce two motivating datasets that are used throughout the article. Section 3 introduces the derivation and interpretation of prediction intervals (regions) following meta‐analysis, in terms of the discrimination ability of a test (sensitivity, specificity and *c* statistic). Section 4 suggests approaches to derive PPV and NPV in new populations and outlines the cross‐validation framework for examining their calibration. Section 5 considers extension to comparing tests. Section 6 provides some discussion, and Section 7 concludes.

## 2 Motivating datasets

### 2.1 Accuracy of ear temperature for diagnosing fever in children

Craig *et al.* 11 systematically reviewed thermometry studies comparing temperatures taken at the ear and rectum in children, and of clinical interest is the accuracy of infrared ear thermometry for diagnosing fever 12. Eleven studies (involving 2323 children) evaluated the accuracy of a ‘FirstTemp’ branded ear thermometer in relation to an electronic rectal thermometer. Rectal temperature was the reference measure, as it is a well‐established method of measuring temperature in children, and it guides clinical decisions and the definition of fever. However, measuring temperature at the ear is clearly more acceptable than measuring temperature at the rectum, and so ear measurements would be preferable if their diagnostic accuracy is adequate. All studies defined patients with an ear temperature ≥38.0 °C to be test positive, and the gold standard definition of fever was a rectal temperature ≥38.0 °C (Table 1), consistent with National Health Service guidelines for diagnosing fever in children at the time of these studies. The studies included children already in hospital or attending accident and emergency 11, and so the observed prevalence of fever was high, around 50%. The summary results for each study are shown in Table 1 in terms of the totals with and without fever, estimated sensitivity and specificity and the number of true positives and negatives. Meta‐analysis is required to summarise test accuracy across these studies, to appropriately quantify any between‐study heterogeneity in test accuracy and – most important to this article – to identify the potential performance of the test in new populations.

**Table 1:** Summary of the 11 temperature studies identified for meta‐analysis; each study used a threshold of 38 °C to define fever, an electronic rectal thermometer and a FirstTemp ear thermometer.

First author	*r*_11*i*_	*n*_1*i*_	Sensitivity	*r*_00*i*_	*n*_0*i*_	Specificity	Observed prevalence
Brennan	150	203	0.74	155	167	0.93	0.55
Davis	9	18	0.50	46	48	0.96	0.27
Green	8	9	0.89	12	12	1.00	0.43
Greenes	53	109	0.49	193	195	0.99	0.36
Hoffman	30	42	0.71	56	58	0.97	0.42
Hooker	10	15	0.67	24	24	1.00	0.38
Lanham	53	103	0.51	74	75	0.99	0.58
Muma	48	87	0.55	136	136	1.00	0.39
Nypaver	282	425	0.66	445	453	0.98	0.48
Rhoads	7	27	0.26	38	38	1.00	0.42
Stewart	57	59	0.97	20	20	1.00	0.75

^r^11i, number of true positives; *n*_1*i*_, number with fever.

^r^00i, number of true negatives; *n*_0*i*_, number without fever.

### 2.2 Accuracy of % change in PTH for predicting risk of hypocalcaemia

As mentioned, Noordzij *et al.* 7 examined the accuracy of the % change in PTH for identifying patients at high risk of becoming hypocalcemic within 48 h after a thyroidectomy. The % change from baseline (rather than absolute change) was used to minimise the impact of different methods of measuring PTH across studies. We focus here on the accuracy of PTH when using a threshold of 65%, above which a patient is classed as test positive. Table 2 summarises the test accuracy results for five cohort studies for % change in PTH when measured from baseline (before surgery) to 0–20 min post‐surgery. Similarly, Table 3 summarises the five cohort studies evaluating % change in PTH when measured from baseline to 1–2 h post‐surgery. Meta‐analysis results are important here to inform whether PTH can suitably identify those at high risk of hypocalcaemia (to ensure they remain in hospital) and those at low risk of hypocalcaemia (who may therefore benefit from early hospital discharge). If PTH appears useful, understanding how best to translate the meta‐analysis results into clinical practice is then crucial.

**Table 2:** Summary of five cohort studies evaluating the accuracy of a >65% change in PTH (measured pre‐operation to 0–20 min post‐thyroidectomy) for identifying hypocalcaemia.

First author	*r*_11*i*_	*n*_1*i*_	Sensitivity	*r*_00*i*_	*n*_0*i*_	Specificity	Observed proportion with outcome
Lo	11	11	1.0	56	89	0.63	0.11
Lombardi	13	16	0.81	31	35	0.89	0.31
McLeod	9	13	0.69	33	43	0.77	0.23
Warren, 2002	3	4	0.75	10	12	0.83	0.25
Warren, 2004	2	3	0.67	20	23	0.87	0.12

^r^11i, number of true positives; *n*_1*i*_, number with hypocalcaemia.

^r^00i, number of true negatives; *n*_0*i*_, number without for identifying hypocalcaemia.

PTH, parathyroid.

**Table 3:** Summary of five cohort studies evaluating the accuracy of a >65% change in PTH (measured pre‐operation to 1–2 h post‐thyroidectomy) for identifying hypocalcaemia.

First author	*r*_11*i*_	*n*_1*i*_	Sensitivity	*r*_00*i*_	*n*_0*i*_	Specificity	Observed proportion with outcome
Lam	12	12	1.0	24	26	0.92	0.32
Lombardi	15	16	0.94	29	35	0.83	0.31
McLeod	11	12	0.92	19	25	0.76	0.32
Warren, 2002	1	2	0.50	6	8	0.75	0.2
Warren, 2004	3	3	1.0	17	23	0.74	0.12

^r^11i, number of true positives; *n*_1*i*_, number with hypocalcaemia.

^r^ 00i, number of true negatives; *n*_0∖*i*_, number without hypocalcaemia.

PTH, parathyroid.

## 3 Examining and summarising the discrimination of a test using meta‐analysis

*Discrimination* statistics, such as sensitivity and specificity, quantify how a test distinguishes between patients with and without the disease (or outcome) of interest and are thus conditional on knowing true disease status. In this section, we show how to use meta‐analysis results for predicting a test's discrimination performance in a new population. For simplicity, we refer only to *diagnostic* tests, but the same principles apply for prognostic test studies where outcome status is known for all patients by a given time.

### 3.1 Sensitivity and specificity

#### 3.1.1 Bivariate meta‐analysis model

Suppose there are *k* test accuracy studies (indexed by *i*, where *i*= 1 to *k*) including *n*_1*i*_ and *n*_0*i*_ patients with and without disease, respectively. The test classifies each patient as either positive or negative, with the aim that those positive are truly diseased and those negative are truly non‐diseased. Summarising test results over all patients in each study produces aggregate data in the form of *r*_11*i*_ (true positives), the number of patients in study *i* with a positive test result who truly have the disease, and *r*_00*i*_ (true negatives), the number of patients in study *i* with a negative test result who truly do not have the disease (true negatives). The observed sensitivity in each study is *r*_11*i*_/*n*_1*i*_, and the observed specificity is *r*_00*i*_/*n*_0*i*_. In this situation, a bivariate random‐effects meta‐analysis can jointly synthesise sensitivity and specificity across studies 2, 3, with *r*_11*i*_ and *r*_00*i*_ modelled directly using the binomial distribution 4, 13 and allowing for potential between‐study heterogeneity in logit‐sensitivity and logit‐specificity, and their potential between‐study correlation due to explicit and implicit differences in threshold value across studies 2.

(1) $$\begin{array}{c} r_{11i}∼\mathrm{Binomial}\left(n_{1i},p_{1i}\right)\\ r_{00i}∼\mathrm{Binomial}\left(n_{0i},p_{0i}\right)\\ \left(\begin{array}{l} \text{logit}\left(p_{1i}\right)\\ \text{logit}\left(p_{0i}\right) \end{array}\right)\;\;∼N\left[\left(\begin{array}{l} \beta_{1}\\ \beta_{0} \end{array}\right)\;\;,\;\Omega \right]\;\;,\;\Omega =\left(\begin{array}{ll} \tau_{1}^{2} & \tau_{1}\tau_{0}\rho \\ \tau_{1}\tau_{0}\rho & \tau_{0}^{2} \end{array}\right) \end{array}$$

Model (1) specifies an underlying logit‐sensitivity and logit‐specificity in each study, by logit(*p*_1*i*_) and logit(*p*_0*i*_), respectively, which are assumed normally distributed about a mean logit‐sensitivity of *β*_1_ and a mean logit‐specificity of *β*_0_, with between‐study variances $\tau_{1}^{2}$ and $\tau_{0}^{2}$, respectively, and between‐study correlation *ρ*. Estimation of model (1) is possible in both frequentist and Bayesian frameworks, using maximum likelihood estimation via Gaussian quadrature 4, 14, 15, 16 or MCMC estimation using Gibbs sampling, respectively 17, 18, 19, 20. For brevity, we present full estimation details in the Supporting Information (S1 and S2).

#### 3.1.2 Summarising discrimination for clinical practice

When reporting results from model (1), researchers typically focus on the summary estimates of sensitivity and specificity, obtained by exp(${\hat{\beta }}_{1})$/[1+ exp(${\hat{\beta }}_{1})$] and exp(${\hat{\beta }}_{0})$/[1+ exp(${\hat{\beta }}_{0})$], respectively, and their CIs (or joint confidence region) 2. However, because of heterogeneity, these summary results may be misleading as they do not necessarily reflect the test's sensitivity and specificity when applied to particular populations. We therefore suggest that researchers should also calculate and report

- a prediction region for the true sensitivity and specificity in a new population 2, such as a 95% prediction region; and
- the probability that the true sensitivity and specificity will *both* be above some clinically acceptable values in the new population.

These measures reveal the potential impact of heterogeneity on discrimination performance in new populations and thereby help indicate whether a test is fit for purpose. For example, if both sensitivity and specificity need to be at least 90% for the test to be considered useful, there should be a large probability that sensitivity and specificity will both meet this criteria in a new population. To make these inferences following estimation of model (1), the joint predictive distribution for a new pair of sensitivity and specificity needs to be derived. If *β*_1_, *β*_0_, $\tau_{1}^{2}$, $\tau_{0}^{2}$ and *ρ* were known, then this distribution on the logit‐scale would be

(2) $$\left(\begin{array}{l} \text{logit}{\left(p_{1i}\right)}_{\mathrm{NEW}}\\ \text{logit}{\left(p_{0i}\right)}_{\mathrm{NEW}} \end{array}\right)\;\;∼\;N\;\;\left[\left(\begin{array}{l} \beta_{1}\\ \beta_{0} \end{array}\right)\;\;,\;\Omega \right]\;\;,\;\Omega =\left(\begin{array}{ll} \tau_{1}^{2} & \tau_{1}\tau_{0}\rho \\ \tau_{1}\tau_{0}\rho & \tau_{0}^{2} \end{array}\right)$$

However, these parameters are not known, and so the predictions should also account for their uncertainty. This is most natural in a Bayesian framework 8, where the uncertainty in all parameters estimated in model (1) is propagated when deriving predictions for new sensitivities and specificities. For our Bayesian application of model (1) in this paper, vague prior distributions of *N*(0,100000) were specified for *β*_1_ and *β*_0_, and a Wishart prior distribution for **Ω** ^−1^ (Supporting Information S2). However, alternative priors for the components of **Ω** are also possible 21, 22. During the Bayesian estimation, posterior inferences about logit(*p*_1*i*_)_*NEW*_ and logit(*p*_1*i*_)_*NEW*_ can made by sampling from their joint posterior (predictive) distribution to calculate 95% intervals and regions, and joint probability statements.

In a frequentist framework, a bivariate *t*‐distribution with *k* − 2 degrees of freedom might be used to approximate the joint predictive distribution, which extends the frequentist use of a univariate *t*‐distribution to derive a prediction interval for a single measure 8, 9. For example, a prediction interval for logit(*p*_1*i*_)_*NEW*_ is

(3) $${\hat{\beta }}_{1}-t_{k-2}\sqrt{{\hat{\tau }}_{1}^{2}+\mathrm{Var}({\hat{\beta }}_{1})},{\hat{\beta }}_{1}+t_{k-2}\sqrt{{\hat{\tau }}_{1}^{2}+\mathrm{Var}({\hat{\beta }}_{1})}$$

where Var(${\hat{\beta }}_{1})$ is the variance of ${\hat{\beta }}_{1}$ and *t*_*k* − 2_ is the 100(1 − *α*/2) percentile of the *t*‐distribution with *k* − 2 degrees of freedom, with *α* usually chosen as 0.05 to give a 95% prediction interval. A *t*‐distribution, rather than a normal distribution, is used to help account for the uncertainty in ${\hat{\tau }}_{1}^{2}$.

### 3.2 *c* Statistic

Tests measured on a continuous scale (such as PTH) allow sensitivity and specificity to be estimated at each of multiple thresholds to define test positive and test negative and thus produce an ROC curve. In this situation, a measure of the test's discrimination across all thresholds is the area under the ROC curve, otherwise known as the *c* statistic 23. This gives the probability that, if one diseased person and one non‐diseased person were chosen at random, the continuous test value would be larger for the diseased person. Given multiple studies, Van Klaveren *et al.* 24 propose that a random‐effects meta‐analysis of the *c* statistic estimates is useful:

(4) $$\begin{array}{c} c_{i}∼N(\theta_{i},s_{i}^{2})\\ \theta_{i}∼N(\mu_{c},\tau_{c}^{2}) \end{array}$$

Here, *c*_*i*_ is the *c* estimate in study *i* assumed to have an (approximate) normal sampling distribution with its variance, $s_{i}^{2}$, assumed known and its mean, *θ*_*i*_, the true *c* statistic in study *i*, which itself is assumed drawn from a normal distribution with summary mean *μ*_*c*_ and between‐study variance $\tau_{c}^{2}$. When the raw test data are available from each study, the *c*_*i*_ can easily be obtained using standard statistical packages, and most typically, bootstrapping can be used to obtain the $s_{i}^{2}$ required. Model (4) can be estimated in a frequentist framework, for example, using methods of moments or restricted maximum likelihood, and the 95% prediction interval is then derived using

(5) $${\hat{\mu }}_{c}-t_{k-2}\sqrt{{\hat{\tau }}_{c}^{2}+\mathrm{Var}({\hat{\mu }}_{c})},{\hat{\mu }}_{c}+t_{k-2}\sqrt{{\hat{\tau }}_{c}^{2}+\mathrm{Var}({\hat{\mu }}_{c})}$$

where Var(${\hat{\mu }}_{c})$ is the variance of ${\hat{\mu }}_{c}$ and other terms follow as outlined in Equation (3). In a Bayesian framework, (vague) prior distributions are required, such as *μ*_*c*_∼*U*(0.5,1) and *τ*_*c*_∼*U*(0,0.25), and then probability statements about the potential *c* statistic in a new population can obtained from the posterior distribution for *θ*_*iNEW*_, such as the probability that the *c* statistic will be above 0.9.

### 3.3 Application

#### 3.3.1 Ear temperature for diagnosis of fever

**Figure 1:**
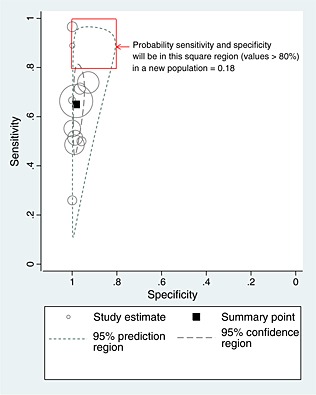
Confidence and prediction regions following application of model (1) to the temperature data.

Model (1) was fitted to the 11 studies evaluating an ear temperature ≥38.0 °C for identifying fever in children (Table 1). The frequentist results are displayed in Figure 1 and are very similar to the results from a Bayesian approach, which we now discuss. The summary sensitivity is 0.65 (95% CrI: 0.51 to 0.77) and the summary specificity is 0.98 (95% CrI: 0.96 to 0.99). The summary specificity is high, so those without fever are usually correctly classed as negative (<38.0 °C). However, the summary sensitivity is low, indicating that many children with fever are not classed as positive (≥38.0 °C). Furthermore, despite the same ear thermometer manufacturer (firsttemp), a consistent threshold (38.0 °C) and a common reference standard (rectal temperature), there is considerable between‐study heterogeneity in both sensitivity and specificity. This is illustrated by the wide prediction region within Figure 1. A 95% prediction interval for sensitivity in a new population is calculated as 0.21 to 0.94 and for specificity is calculated as 0.89 to 1. Direct probabilistic statements can also be made from the (joint) predictive distribution for a new sensitivity and specificity (Supporting Information S3). For example, in a new population, the probability that sensitivity will be over 80% is 0.19, and the probability that specificity will be over 80% is 0.99. Further, the joint probability that in a new population *both* sensitivity and specificity will be >80% is just 0.19 (Figure 1). Hence, if sensitivity and specificity above 80% are deemed the minimum acceptable, there is less than a one in five chance that ear temperature will be adequate within a new population. So there is little evidence here to suggest that ear temperature can replace rectal temperature as an acceptable technique for assessing fever.

#### 3.3.2 PTH for predicting hypocalcaemia

Parathyroid is measured on a continuous scale, and as the raw data were available to us, we calculated the *c* statistic and its 95% CI and bootstrap standard error for each of the five studies. Model (4) was then estimated using methods of moments 25, and the results are shown in the Supporting Information (S4) for the test relating to the % change in PTH from baseline to 0–20 min post‐surgery. The summary *c* statistic is encouragingly high (0.92, 95% CI: 0.86 to 0.97). Furthermore, although there is heterogeneity in discrimination performance across studies (*I* ^2^=28*%*), the 95% prediction interval derived from Equation (5) indicates that *c* in a new population will be between 0.78 and 1.00, and is thus also predominately high. Bayesian inferences were similar, but the width of intervals were sensitive to the prior distribution for the between‐study variance. However, for all the priors considered, there was a probability of about 0.89 that *c* in a new population will be above 0.8 and a probability of 0.59 that it will be above 0.9. Therefore, there is large probability that PTH measured at 0–20 min will have very good discrimination when applied in new populations. The discrimination was similar when PTH was measured 1–2 h post‐surgery.

For implementation of PTH in clinical practice, a single threshold would be chosen to define test positive and negative. Noordzij *et al.* 7 recommend a threshold of a 65% change in PTH from pre‐surgery to 1–2 h post‐surgery, for which Bayesian estimation of model (1) gives a summary sensitivity of 0.93 (95% CrI: 0.83 to 0.99) and a summary specificity of 0.81 (95% CrI 0.71 to 0.89). The probability that sensitivity will be greater than 0.8 in a new population is 0.96, and for specificity it is 0.59 (Supporting Information S3). The probability that both will be above 0.8 is 0.57. In other words, we expect that in nearly 6 out of 10 populations, both the sensitivity and specificity of the PTH test will be above 0.8. Thus, PTH measured at 1–2 h appears to have high discrimination for potential clinical use, and this is evaluated further in terms of post‐test predictions in Section 4.3.

## 4 Examining and summarising the calibration of post‐test probabilities derived following meta‐analysis

In practice, clinicians are more interested in PPV and NPV, the post‐test disease (outcome) probabilities conditional on knowing the test result. Following meta‐analysis, an explicit strategy (or statistical equation) is therefore needed to tell clinicians how to derive PPV and NPV in their population. Further, any recommended strategy or equation should be formally evaluated in new data, a concept known as *external validation* in the prediction modelling field 26. Thus, in this section, we consider (i) strategies for deriving PPV and NPV in new populations using existing meta‐analysis results, and then (ii) an approach called internal–external cross‐validation 27, 28, which allows the strategies to be examined and compared in terms of calibration of predicted and observed PPV and NPV.

### 4.1 Strategies to derive PPV and NPV in new populations

For simplicity, let us focus on prognostic test studies, as these are usually cohort studies for which absolute risk can be observed 29. Let there be *n*_1*i*_ and *n*_0*i*_ patients that do and do not have an outcome by a particular time, respectively, and let *r*_11*i*_ be the number of patients in study *i* who had the outcome and a positive test result at baseline, and *r*_00*i*_ be the number of patients in study *i* who did not have an outcome and had a negative test result at baseline. We now suggest three possible strategies for deriving PPV and NPV in a new population (*L*), given *k* existing studies for meta‐analysis.

#### 4.1.1 Modelling option A: use summary values of sensitivity, specificity and prevalence

The simplest approach is to derive the new PPV and NPV by first obtaining meta‐analysis summary estimates of sensitivity, specificity and prevalence, and then plugging them into the well‐known Bayes theorem solutions for deriving PPV and NPV from sensitivity, specificity and prevalence:

(6) $${\text{PPVnew}}_{L}=\frac{{\text{sensitivity}}_{\text{MA}}\times {\text{prevalence}}_{\text{MA}}}{\left[{\text{sensitivity}}_{\text{MA}}\times {\text{prevalence}}_{\text{MA}}]+[(1-{\text{specificity}}_{\text{MA}})(1-{\text{prevalence}}_{\text{MA}})\right]}$$

(7) $${\text{NPVnew}}_{L}=\frac{{\text{specificity}}_{\text{MA}}\times (1-{\text{prevalence}}_{\text{MA}})}{\left[(1-{\text{sensitivity}}_{\text{MA}})\times {\text{prevalence}}_{\text{MA}}]+[{\text{specificity}}_{\text{MA}}\times (1-{\text{prevalence}}_{\text{MA}})\right]}$$

In these equations, ‘MA’ denotes the summary meta‐analysis value, and ‘new’ denotes the predicted value in the new population, *L*. This approach mimics situations where nothing is known about test accuracy performance and prevalence in the new population, and so one simply takes the summary values from existing studies. The summary sensitivity and summary specificity can be obtained from fitting model (1) to the available studies (which may include cohort and case‐control studies), and the summary prevalence can be obtained from a meta‐analysis of available cohort studies, for example, by using maximum likelihood to fit:

(8) $$\begin{array}{c} n_{1i}∼\mathrm{Binomial}\;\left(N_{i},{\text{prevalence}}_{i}\right)\\ \text{logit}({\text{prevalence}}_{i})∼N\left(\alpha ,\tau_{\alpha }^{2}\right) \end{array}$$

where *N*_*i*_ is the total sample size in study *i* and the summary prevalence is obtained by $\exp(\hat{\alpha })/(1+\exp(\hat{\alpha }))$.

#### 4.1.2 Modelling option B: use summary values of sensitivity and specificity, and known prevalence in the intended population

Even when there is no between‐study heterogeneity in sensitivity and specificity, it is likely that there is heterogeneity in the prevalence. Thus, PPV and NPV can be tailored to particular populations by adapting Equations (6) and (7) by including the prevalence in the new population of interest:

(9) $${\text{PPVnew}}_{L}=\frac{{\text{sensitivity}}_{\text{MA}}\times {\text{prevalence}}_{L}}{\left[{\text{sensitivity}}_{\text{MA}}\times {\text{prevalence}}_{L}]+[(1-{\text{specificity}}_{\text{MA}})(1-{\text{prevalence}}_{L})\right]}$$

(10) $${\text{NPVnew}}_{L}=\frac{{\text{specificity}}_{\text{MA}}\times (1-{\text{prevalence}}_{L})}{\left[(1-{\text{sensitivity}}_{\text{MA}})\times {\text{prevalence}}_{L}]+[{\text{specificity}}_{\text{MA}}\times (1-{\text{prevalence}}_{L})\right]}$$

where prevalence_*L*_ is the known prevalence in the intended population. If this prevalence is unknown, it might be substituted by the observed prevalence from a sample of patients from the intended population, to use as a proxy for the true prevalence.

#### 4.1.3 Modelling option C: fit a bivariate meta‐analysis of PPV and NPV, with prevalence as a covariate

Rather than deriving PPV and NPV by considering sensitivity and specificity independent of prevalence, a more sophisticated option is to consider sensitivity, specificity and prevalence jointly. Chu *et al.* 30 propose a trivariate meta‐analysis for this purpose. However, in our experience, it is difficult to estimate reliably the two between‐study correlations in this model. Thus, we take the approach suggested by Leeflang *et al.* 5, where PPV and NPV (rather than sensitivity and specificity) are now the two measures of interest, and a covariate included for prevalence. This bivariate meta‐regression is applied to the available cohort studies in the meta‐analysis and can be written as

(11) $$\begin{array}{c} r_{11i}∼\mathrm{Binomial}\;\left(n_{+i},\mathrm{PP}V_{i}\right)\\ r_{00i}∼\mathrm{Binomial}\;\left(n_{-i},\mathrm{NP}V_{i}\right)\\ \left(\begin{array}{l} \text{logit}\left(\mathrm{PP}V_{i}\right)\\ \text{logit}\left(\mathrm{NP}V_{i}\right) \end{array}\right)\;\;∼\;N\;\;\left[\left(\begin{array}{l} \delta_{1}+\gamma_{1}{\text{prevalence}}_{i}\\ \delta_{0}+\gamma_{0}{\text{prevalence}}_{i} \end{array}\right)\;,\;\Omega \right]\;,\;\Omega =\left(\begin{array}{ll} \tau_{\mathrm{PPV}}^{2} & \tau_{\mathrm{PPV}}\tau_{\mathrm{NPV}}\rho \\ \tau_{\mathrm{PPV}}\tau_{\mathrm{NPV}}\rho & \tau_{\mathrm{NPV}}^{2} \end{array}\right) \end{array}$$

Here, *n*_+*i*_ and *n*_−*i*_ denote the number who tested positive and negative, respectively, in study *i*. The model specifies that the true logit(*PPV*_*i*_) and logit(*NPV*_*i*_) in each study depends on the prevalence (prevalence_*i*_) of that study (note that an alternative specification could be to use logit(prevalence_*i*_) as the covariate, although for brevity, this is not considered further here). It allows for unexplained between‐study heterogeneity in logit(*PPV*_*i*_) and logit(*NPV*_*i*_), $\tau_{\mathrm{PPV}}^{2}$ and $\tau_{\mathrm{NPV}}^{2}$, respectively, and their potential between‐study correlation (*ρ*). A disadvantage of model (11) is that it discards studies (e.g. case‐control studies) that do not allow reliable estimation of PPV and NPV but could otherwise contribute towards sensitivity and specificity.

Model (11) can be fitted using the same techniques as described for model (1), and the estimated parameters enable PPV and NPV to be derived in a new population (*L*) if the prevalence (prevalence_*L*_) in the intended population is specified, as follows:

(12) $${\text{PPVnew}}_{L}=\frac{\exp({\hat{\delta }}_{1}+{\hat{\gamma }}_{1}{\text{prevalence}}_{L})}{1+\exp({\hat{\delta }}_{1}+{\hat{\gamma }}_{1}{\text{prevalence}}_{L})}$$

(13) $${\text{NPVnew}}_{L}=\frac{\exp({\hat{\delta }}_{0}+{\hat{\gamma }}_{0}{\text{prevalence}}_{L})}{1+\exp({\hat{\delta }}_{0}+{\hat{\gamma }}_{0}{\text{prevalence}}_{L})}$$

### 4.2 Internal–external cross‐validation for checking calibration of predicted PPV and NPV in new populations

Whatever strategy is used to derive PPV and NPV in new populations, external validation is required to check the calibration of predictions with observed risk. However, it is well known that calibration performance of predictive probabilities is optimistic when they are estimated and then tested on the same data 26, 31, 32. Thus, to validate the chosen strategy, we suggest using internal–external cross‐validation, which omits a study from the meta‐analysis that derives the prediction equations, so that calibration can be checked in independent data. The process is then repeated, with a different study omitted in each cycle. The approach was proposed by Royston *et al.* 27 and recently extended by Debray *et al.* 28 for validation of multivariable prediction models. We now implement it within the context of test accuracy studies and extend it to include a meta‐analysis that summarises the calibration performance across all cycles.

#### 4.2.1 Summarising overall calibration performance

Suppose that the focus is on summarising the *overall* calibration between predicted and observed outcome risks in new populations. The internal–external cross‐validation approach proceeds as follows:

1. Select a study *L* to be omitted
2. In the remaining *k* − 1studies, fit a chosen meta‐analysis model and use a chosen strategy (e.g. options A to C earlier) to produce equations for deriving PPV and NPV in a new population. Apply these equations to the omitted study *L*, to calculate the predicted PPVnew_*L*_and NPVnew_*L*_.
3. In the omitted study *L*, calculate the total observed (O) and the total expected (E) outcomes, where
  $$O_{L}=n_{1L}$$
  (14) $$E_{L}={\text{PPVnew}}_{L}(r_{11L}+(n_{0L}-r_{00L}))+(1-{\text{NPVnew}}_{L})(r_{00L}+(n_{1L}-r_{11L}))$$
  where PPVnew_*L*_and NPVnew_*L*_are those calculated using the equations derived in step (2).
4. In the omitted study, compute the *overall calibration* of predicted probabilities by (O_*L*_ */*E_*L*_) = (O*/*E)_*L*_, and calculate ln(O*/*E)_*L*_ and its standard error, s_*L*_.
  Defining P_O*L*_=O_*L*_/(*n*_1*L*_+*n*_0*L*_) and P_E*L*_=E_*L*_/(*n*_1*L*_+*n*_0*L*_), we can write

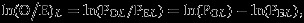

with P_O*L*_ and P_E*L*_ the observed and expected (O/E) proportion with the outcome in the omitted study, respectively. Treating the expected proportion P_E*L*_ as fixed, the standard error of ln(O*/*E)_*L*_equals the standard error of ln(P_O*L*_), which is given approximately by 33
  (15) $$s_{L}=\sqrt{\frac{1-P_{OL}}{(n_{1L}+n_{0L})P_{\mathrm{OL}}}}$$
5. Repeat steps (1) to (4) for each omitted study, giving a set of *k* values for ln(O*/*E)_*L*_ and s_*L*_.
6. Perform a (frequentist or Bayesian) random‐effects meta‐analysis of the ln(O*/*E)_*L*_values as follows:
  (16) $$\begin{array}{c} {\text{ln(O/E)}}_{L}∼N\left(\theta_{L},s_{L}^{2}\right)\\ \theta_{L}∼N\left(\beta ,\tau^{2}\right) \end{array}$$
7. Summarise calibration across all studies by displaying the meta‐analysis results using a forest plot including the summary O/E value, its 95% CI, *I* ^2^ and a 95% prediction interval for the O/E value in a new population.

The omitted studies in step (1) must be cohort studies (or cross‐sectional studies for diagnostic tests). Other studies, such as case‐control studies, that do not provide unbiased estimates of outcome risk (or disease prevalence for diagnostic test studies) might contribute to the developed prediction equations in step (2) (e.g. using options A or B in Section 4.1).

Compared with previous use of the internal–external cross‐validation approach 27, 28, we have added the meta‐analysis component in steps (6) and (7). Because uncertainty in each ratio O/E reflects only within‐study error for the omitted study and not the error associated with computing the expected values from the other studies, it is appropriate to combine these across studies using the within‐study errors in the weights. This allows us to quantify whether observed differences in overall calibration are due to chance or heterogeneity and to summarise calibration performance on average and in potential new populations. Perfect calibration would be revealed by a summary O/E value of 1 (indicating perfect calibration on average across all populations), together with no between‐study heterogeneity in calibration performance, resulting in a narrow 95% prediction interval for the O/E in a new population. Such perfection is unlikely, and so researchers might consider what calibration performance would be acceptable. For example, a 95% prediction interval for O/E falling between 0.9 and 1.1 might be deemed acceptable, as the true risk is then within 10% of the predicted risk in new populations. In other words, one desires a large probability (0.95 or more) that O/E is between 0.9 and 1.1 in new population.

#### 4.2.2 Summarising calibration of PPV and NPV separately and more exactly

The earlier analyses allow the *overall* calibration to be examined, comparing the total observed outcome events across both test‐positive and test‐negative individuals with the total expected number. Also of interest is the calibration of PPV and NPV themselves, especially as one might be more clinically important than the other. For example, in the thyroidectomy example, a reliable NPV is crucial as those with a negative PTH test may be discharged early to release valuable resources (such as beds and clinical care) to the hospital. The calibration of PPV and NPV can be undertaken using the same approach as described in Section 4.2.1 but with a separate O/E estimated for test‐positive and test‐negative patients in each study, followed by a separate meta‐analysis of O/E for each.

However, when considering NPV (or PPV) separately, it is increasingly likely that some studies will have no events (or no non‐events) for test‐negative (or test‐positive) patients. In such studies, the standard error of ln(O*/*E)_*L*_ cannot be derived using Equation (15) as P_O*L*_ is 1 (or 0), and thus, meta‐analysis model (16) is problematic. A more exact meta‐analysis is thus proposed, which models the binomial likelihood directly in each study to avoid having to derive standard errors and thereby accommodate studies with zero events (or zero non‐events) 13. Without loss of generality, consider just PPV so that the analysis includes only test‐positive (‘+’) patients. The model is written as

(17) $$\begin{array}{c} r_{11L}∼\mathrm{Binomial}\;\left(n_{+L},\mathrm{PP}V_{L}\right)\\ \text{logit}(\mathrm{PP}V_{L})=\alpha_{L}+\text{logit}\;\left({\text{PPVnew}}_{L}\right)\\ \alpha_{L}∼\text{N}\;\left(a,\tau_{\alpha }^{2}\right) \end{array}$$

where logit(PPVnew_*L*_) is an offset term corresponding to the logit of the predicted PPV for study *L* obtained from step (2). *n*_+*L*_ is the total number of test‐positive patients in study *L*, and *r*_11*L*_ is the number of those who truly have the outcome. The *α*_*L*_ are often referred to as the calibration‐in‐the‐large 26, and so the estimate of *a* provides the summary calibration‐in‐the‐large. A more formal interpretation is that *a* is the average log odds ratio, summarising the log of observed to expected odds across studies. A value of 0 thus indicates perfect calibration. The heterogeneity, $\tau_{\alpha }^{2}$, is the between‐study variation in calibration, and this will be zero if calibration performance is consistent across all populations. Model (17) can be fitted in either a Bayesian or a frequentist framework (Supporting Information S5 and S6), with posterior (predictive) inferences then derived. For example, following frequentist estimation and given a particular PPVnew_*L*_, an approximate 95% prediction interval for the true logit PPV is

(18) $$\begin{array}{c} \left(\hat{a}-t_{k-2,0.975}\sqrt{{\hat{\tau }}_{\alpha }^{2}+\mathrm{Var}(\hat{a})}\right)+\text{logit}\;\left({\text{PPVnew}}_{L}\right),\left(\hat{a}+t_{k-2,0.975}\sqrt{{\hat{\tau }}_{\alpha }^{2}+\mathrm{Var}(\hat{a})}\right)+\text{logit}\;\left({\text{PPVnew}}_{L}\right) \end{array}$$

The lower and upper bounds can then be transformed back to the PPV scale, or the O/E scale, as desired.

### 4.3 Applications

**Figure 2:**
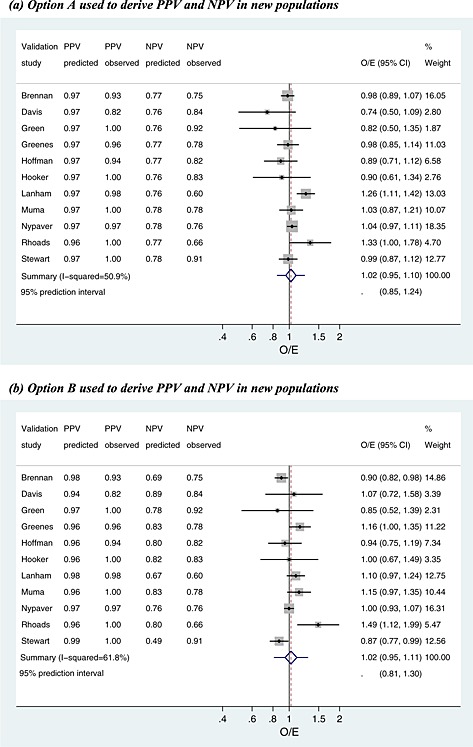
Meta‐analysis of the observed/expected (O/E) calibration statistics (frequentist estimation of model (16)) from the internal–external cross‐validation approach applied to the ear temperature data for diagnosis of fever. PPV, positive predictive value; NPV, negative predictive value.

**Figure 3:**
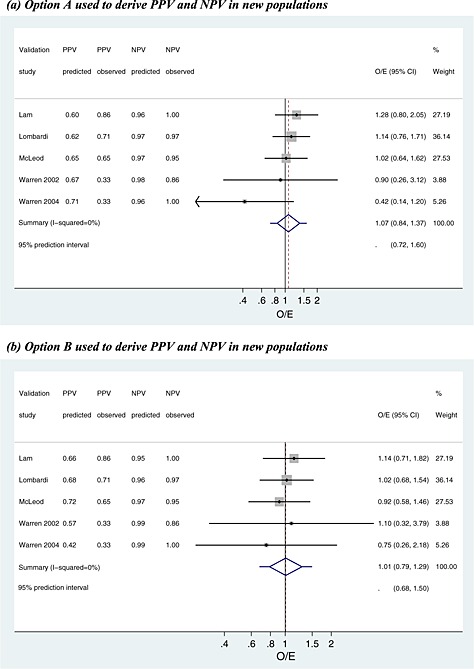
Meta‐analysis of the observed/expected (O/E) calibration statistics (frequentist estimation of model (16)) from the internal–external cross‐validation approach applied to the parathyroid data at 1–2 h for prediction of hypocalcaemia. PPV, positive predictive value; NPV, negative predictive value.

**Figure 4:**
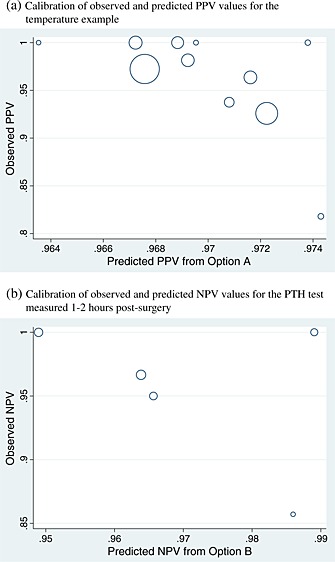
Calibration of predicted and observed post‐test probabilities, for (a) positive predictive value (PPV) derived using option A in the temperature example and (b) negative predictive value (NPV) derived using option B in the parathyroid example. Each circle represents a study and is proportional to the study sample size.

**Figure 5:**
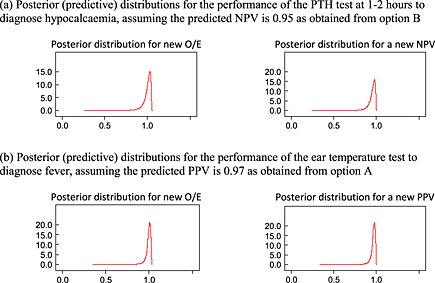
Posterior distributions for the true observed/expected (O/E), positive predictive value (PPV) or negative predictive value (NPV) in a new population, derived from Bayesian estimation of model (17).

We now apply the internal–external cross‐validation approach to the two examples introduced in Section 2. The results are summarised in Tables 4, 5, 6, 7, and Figures 2, 3, 4, 5.

**Table 4:** Results of the internal–external cross‐validation procedure for the temperature data.

Study	Observed no. with fever (O)	Observed prevalence	Summary prevalence from meta‐analysis model (8), ∗	Summary sensitivity from meta‐analysis model (1), ∗	Summary specificity from meta‐analysis model (1), ∗	Option A Post‐test predictions obtained using summary sensitivity, summary specificity and summary prevalence (Equations (6) and (7))				Option B Post‐test predictions obtained using summary sensitivity, summary specificity and observed prevalence (Equations (9) and (10))				Option C Post‐test predictions obtained after bivariate meta‐regression of PPV and NPV on prevalence (Equations (12) and (13))			
						Predicted PPV	Predicted NPV	Expected no. with fever ^ (E)	O/E	Predicted PPV	Predicted NPV	Expected no. with fever ^ (E)	O/E	Predicted PPV	Predicted NPV	Expected no. with fever ^(E)	O/E
Brennan	203	0.55	0.45	0.644	0.99	0.97	0.77	207.59	0.98	0.98	0.69	226.11	0.90	0.98	0.75	211.83	0.96
Davis	18	0.27	0.48	0.661	0.98	0.97	0.76	24.41	0.74	0.94	0.89	16.85	1.07	0.96	0.78	22.71	0.79
Green	9	0.43	0.46	0.631	0.98	0.97	0.76	10.92	0.82	0.97	0.78	10.58	0.85	0.96	0.76	10.80	0.83
Greenes	109	0.36	0.46	0.67	0.98	0.97	0.77	110.91	0.98	0.96	0.83	94.12	1.16	0.95	0.78	106.11	1.03
Hoffman	42	0.42	0.47	0.645	0.99	0.97	0.77	47.23	0.89	0.96	0.80	44.56	0.94	0.97	0.76	47.06	0.89
Hooker	15	0.38	0.46	0.65	0.98	0.97	0.76	16.63	0.90	0.96	0.82	14.97	1.00	0.96	0.78	16.07	0.93
Lanham	103	0.58	0.47	0.666	0.98	0.97	0.76	81.98	1.26	0.98	0.67	94.01	1.10	0.98	0.76	82.71	1.25
Muma	87	0.39	0.46	0.662	0.98	0.97	0.78	84.73	1.03	0.96	0.83	75.98	1.15	0.95	0.78	84.66	1.03
Nypaver	425	0.48	0.45	0.652	0.98	0.97	0.78	408.91	1.04	0.97	0.76	426.06	1.00	0.97	0.76	421.87	1.01
Rhoads	27	0.42	0.47	0.683	0.98	0.96	0.77	20.25	1.33	0.96	0.80	18.12	1.49	0.96	0.77	19.78	1.36
Stewart	59	0.75	0.43	0.65	0.98	0.97	0.78	59.88	0.99	0.99	0.49	67.79	0.87	0.98	0.56	65.61	0.90

∗ Obtained from meta‐an the study in the row.

^ Expected number for the study in the row, based on using Equation (14) and the positive predictive value (PPV) and negative predictive value (NPV) in the prior two columns.

**Table 5:** Meta‐analysis results for overall calibration (O/E) when using model (16), as estimated in a frequentist or Bayesian framework.^1^

Example	Predicted PPV and NPV obtained using…	Statistical framework	Summary O/E (95% CI)	$\hat{\tau }$ (95% CI)	95% prediction interval for O/E in a new population	Probability 0.9 < O/E < 1.1 in a new population
Temperature data	Option A	Bayesian	1.02 (0.93 to 1.11)	0.10 (0.01 to 0.23)	0.78 to 1.31	0.67
	Option A	Frequentist	1.02 (0.95 to 1.10)	0.08	0.85 to 1.24	—
PTH data 1–2 h	Option B	Bayesian	1.02 (0.73 to 1.38)	0.14 (0.01 to 0.45)	0.56 to 1.74	0.40
	Option B	Frequentist	1.01 (0.79 to 1.29)	0	0.68 to 1.50	—

1 All frequentist analyses used method of moments to estimate the model.

All Bayesian analyses used a prior *N*(0, 1 000 000) for the mean ln(O/E) and a prior uniform(0, 0.25) for *τ*, with a 10 000 burn‐in followed by 100 000 samples for posterior inferences.

O/E, observed/expected; PPV, positive predictive value; NPV, negative predictive value.

**Table 6:** Meta‐analysis results for calibration of either PPV or NPV, when using meta‐analysis model (17) as estimated in a frequentist or Bayesian framework.^1^

Example	Meta‐analysis method	Statistical framework	Summary calibration, *a* (95% CI)	${\hat{\tau }}_{a}$	95% prediction interval for O/E in a new population∗	Probability 0.9 < O/E < 1.1 in a new population∗
Calibration for just NPV					
PTH data 1–2 h	Option B	Bayesian	0.24 (−0.97 to 1.81)	0.51 (0.03 to 0.98)	0.86 to 1.05	0.95
	Option B	Frequentist	0.093 (−1.06 to 1.25)	0	0.87 to 1.03	—
PTH data 0–20 min	Option B	Bayesian	0.021 (−0.82 to 1.02)	0.51 (0.03 to 0.97)	0.86 to 1.04	0.95
	Option B	Frequentist	−0.044 (−0.83 to 0.74)	0.34	0.80 to 1.03	—
Calibration for just PPV					
Temperature data	Option A	Bayesian	−0.017 (−0.63 to 0.77)	0.65 (0.11 to 0.99)	0.90 to 1.03	0.98
	Option A	Frequentist	0.015 (−0.75 to 0.78)	0.70	0.87 to 1.03	—

1 All frequentist analyses used maximum likelihood estimation of model (17). All Bayesian analyses used a prior distribution of *N*(0, 1 000 000) for *a*, and a prior distribution of uniform(0, 1) for *τ*, with a 10 000 burn‐in followed by 100 000 samples for posterior inferences. Median values of the posterior distribution are shown for *a* and *τ*.

∗ Based on a predicted positive predictive value (PPV) of 0.97 in the temperature analysis, and a negative predictive value (NPV) of 0.95 in the parathyroid (PTH) analysis.

O/E, observed/expected.

**Table 7:** Results of the internal–external cross‐validation procedure for the PTH data.

Study	Observed no. with fever (O)	Observed prevalence	Summary prevalence from meta‐analysis model (8), *	Summary sensitivity from meta‐analysis model (1), *	Summary specificity from meta‐analysis model (1), *	Option A Post‐test predictions obtained using summary sensitivity, summary specificity and summary prevalence (Equations (6) and (7))				Option B Post‐test predictions obtained using summary sensitivity, summary specificity and observed prevalence (Equations (9) and (10))			
						Predicted PPV	Predicted NPV	Expected no. with outcome^(E)	O/E	Predicted PPV	Predicted NPV	Expected no. with outcome^(E)	O/E
(1) PTH measured at 1–2 h												
Lam	12	0.32	0.26	0.91	0.78	0.60	0.96	9.37	1.28	0.66	0.95	10.51	1.14
Lombardi	16	0.31	0.26	0.93	0.80	0.62	0.97	14.01	1.14	0.68	0.96	15.64	1.02
McLeod	12	0.32	0.26	0.94	0.82	0.65	0.97	11.77	1.02	0.72	0.97	13.11	0.92
Warren, 2002	2	0.20	0.28	0.95	0.82	0.67	0.98	2.21	0.90	0.57	0.99	1.82	1.10
Warren, 2004	3	0.12	0.31	0.93	0.83	0.71	0.96	7.22	0.42	0.42	0.99	3.99	0.75
(2) PTH measured at 0–20 min												
Lo	11	0.11	0.24	0.75	0.83	0.59	0.91	33.60	0.33	0.36	0.96	18.82	0.58
Lombardi	16	0.31	0.16	0.81	0.76	0.39	0.96	8.32	1.92	0.60	0.90	14.12	1.13
McLeod	13	0.23	0.18	0.87	0.81	0.50	0.97	11.15	1.17	0.58	0.95	13.22	0.98
Warren, 2002	4	0.25	0.18	0.83	0.78	0.47	0.95	2.94	1.36	0.56	0.93	3.69	1.08
Warren, 2004	3	0.12	0.21	0.84	0.77	0.49	0.95	3.70	0.81	0.32	0.97	2.26	1.33

* Obtained from meta‐analysing all studies excluding the study in the row.

^ Expected number for the study in the row, based on using Equation (14) and the positive predictive value (PPV) and negative predictive value (NPV) in the prior two columns.

PTH, parathyroid.

#### 4.3.1 Ear temperature for diagnosis of fever

In Section 3, the meta‐analysis revealed that ear temperature above or below 38 °C only moderately discriminated those with and without fever. We now use the internal–external cross‐validation approach to evaluate the calibration of post‐test probabilities, PPV and NPV, derived using options A to C. The results at each cycle of the procedure are given in Table 4 for each strategy, and Figure 2 and Tables 5 and 6 summarise calibration performance.

#### Overall calibration

First consider the overall calibration of observed to expected values, across all patients, for the case where PPV and NPV are derived by option A (use the summary sensitivity, specificity and prevalence from meta‐analysis of the other studies) (Table 4). The predicted PPV and NPV are close to 0.97 and 0.77 for most studies (Table 4). Frequentist estimation of meta‐analysis model (16), using methods of moments, gives a summary O/E close to 1 with a narrow CI (summary O/E = 1.02, 95% CI: 0.95 to 1.10), suggesting excellent calibration on average in new populations (Figure 2(a); Table 5). However, there is heterogeneity in calibration performance (*I* ^2^=51*%*), a likely consequence of the heterogeneity in sensitivity and specificity identified in Section 3. A 95% prediction interval for the O/E in a new population is 0.85 to 1.24, suggesting that the true fever risk will be between 24% higher and 15% lower than that expected in new populations. Bayesian estimation gives similar inferences, although with wider intervals, and there is a probability of 0.66 that 0.9 < O/E < 1.1 (Table 5), and so in two‐thirds of populations, calibration will reassuringly be within 10% of that predicted.

Interestingly, calibration appears worse when the internal–external validation approach is repeated with PPV and NPV calculated using option B (prevalence taken from the omitted study's population, combined with summary sensitivity and specificity). As true population prevalences were unknown in omitted studies, we mimicked this situation by assuming the observed prevalence in the omitted study was actually the true prevalence. The forest plot summarising O/E for this approach is shown in Figure 2(b). Compared with the previous analysis, heterogeneity in calibration performance increases (*I* ^2^=62*%*), and thus, the 95% prediction interval is wider (0.81 to 1.30). Therefore, post‐test predictions appear less reliable using option B rather than option A. Option C (bivariate meta‐regression of PPV and NPV on prevalence) performs similarly to option A. The summary O/E is slightly improved (1.01), but there is additional heterogeneity (*I* ^2^=56*%*), and thus, the 95% prediction interval is similar (0.82 to 1.25).

#### Calibration of PPV

Because of NPV being too low to rule out fever, one might examine just the calibration of PPV. Using option A to derive PPV in new populations, the predicted PPV is close to 0.97 in all cycles of the internal–external cross‐validation approach (Table 4). Some studies did not have any test‐positive children without fever, and thus, the exact meta‐analysis model (17) was used. Maximum likelihood estimation gives a summary calibration, *a*, of 0.015 (95% CI: −0.75 to 0.78) (Table 6). This reveals that, on average, there is close to perfect agreement between expected and observed PPVs in the studies. However, there is heterogeneity (*τ*_*α*_=0.70) in calibration performance, which, when using Equation (18) followed by back transformations, corresponds to a 95% prediction interval of 0.87 to 1.03 for the O/E in a new population where the predicted PPV was 0.97. Bayesian estimates are very similar (Table 6), and there is a large probability of 0.98 that O/E will be between 0.9 and 1.1 in a new population. Further, for a predicted PPV of 0.97, there is a probability of 0.78 that the true PPV will be between 0.95 and 1. The calibration between observed and predicted PPVs using option A is shown in Figure 4(a). Calibration performance is not improved when deriving PPV using options B or C (results not shown), as either the summary calibration performance is worse or the heterogeneity is larger.

Thus, PPV derived from option A appears to calibrate extremely well in new populations. We thus derived a final PPV from option A to be used in clinical practice, obtained by placing into Equation (6) the summary estimates of sensitivity, specificity and prevalence from the meta‐analyses of all 11 studies. This gave a high PPV of 0.97, and thus, an ear temperature ≥38.0 °C strongly indicates that a child truly has a fever.

#### 4.3.2 PTH for predicting hypocalcaemia

Section 3.3 identified that PTH has high sensitivity and specificity at a threshold of 65% when PTH is measured 1–2 h post‐surgery (summary sensitivity, 0.93 and summary specificity, 0.81). For this test, we now use the internal–external cross‐validation approach to examine and compare the calibration of PPV and NPV in new populations as derived from options A and B. Given there were only five studies in the meta‐analysis, the meta‐regression approach of option C was not deemed sensible and is thus not evaluated here.

#### Overall calibration

NPV and PPV are both estimated consistently high during the internal–external cross‐validation process (Table 7). When post‐test probabilities are based on option A (using the summary sensitivity, specificity and prevalence), frequentist estimation of model (16) indicates there is no heterogeneity in calibration performance (*I* ^2^=0*%*) (Figure 3(a)). The summary O/E is 1.07, suggesting under‐prediction of hypocalcaemia events in new populations. When the post‐test probabilities are derived using option B (summary sensitivity and specificity, combined with observed prevalence in omitted study), there continues to be no heterogeneity, but the summary O/E is improved to 1.01, indicating almost perfect calibration on average (Figure 3(b)). Although there is no estimated heterogeneity, the prediction interval (0.68 to 1.50) is wide (Figure 3(b)), as it accounts for large uncertainty in the heterogeneity and pooled estimates, a consequence of only five studies in the meta‐analysis. Bayesian posterior estimates are very similar, although intervals are even wider. There is a probability of only 40% that the true O/E is between 0.9 and 1.1, again reflecting the large uncertainty in the meta‐analysis.

#### Calibration of NPV

As mentioned, the pivotal element for the PTH test is good calibration for NPV so that patients are not wrongly discharged from hospital. Table 7 shows that the predicted NPV is consistently high in the cycles of internal–external cross‐validation, ranging from 0.96 to 0.98 when the summary prevalence is used (option A) and from 0.95 to 0.99 when the observed population prevalence is used (option B). To examine calibration performance of predicted NPV in new populations, meta‐analysis model (17) was applied to accommodate studies that had no test‐negative patients with hypocalcaemia.

For predicted NPV derived using option B, the summary calibration, *a*, is 0.093 following frequentist estimation, which suggests only a small positive miscalibration between observed and predicted NPVs. There is also no estimated heterogeneity in calibration performance. For a predicted NPV of 0.95, these results correspond to a 95% prediction interval for the true NPV in a new population of 0.78 to 0.99, or an O/E of 0.87 to 1.03. These intervals are wide because of the small number of studies. For the same reason, Bayesian estimation is especially sensitive to the choice of prior distribution for *τ*, and frequentist and Bayesian results are less similar here than for the temperature analyses (Table 6). However, the Bayesian approach agrees that option B gives well‐calibrated NPVs in new populations. For example, when using a U(0,1) prior for *τ*, there is a large probability of 0.95 that option B will give a predicted NPV that calibrates within 10% of the true NPV in a new population (Table 6). The calibration of observed and predicted NPVs is shown in Figure 4(b). Predictive (posterior) distributions for the NPV and O/E in a new population are shown in Figure 5. Option A performs very similarly to option B.

Thus, although further studies would be useful to reduce the width of prediction intervals, the current evidence strongly suggests that option B (or even A) will give predicted NPVs that calibrate well in new populations. Using option B and deriving summary sensitivity and specificity from a meta‐analysis of all 5 studies, the final prediction equation for a new NPV i

$${\text{NPVnew}}_{L}=\frac{0.81\times (1-{\text{prevalence}}_{L}\text{)}}{\left[(1-0.93)\times {\text{prevalence}}_{L}]+[0.81\times (1-{\text{prevalence}}_{L})\right]}$$

## 5 Extensions

We now briefly consider two potentially useful extensions of our work.

### 5.1 Comparing test performance

The examination of calibration and discrimination performance in new populations is also a novel way to compare the performance of two different tests, or for comparing the same test at different thresholds or measurement methods. For example, in the PTH example, there are two competing tests: PTH measured at 0–20 min or at 1–2 h. Both have a high discrimination indicated by *c* statistics above 0.80 (see, e.g. Supporting Information S4), but which will perform better in new populations? For illustration, we now evaluate this for a threshold of 65%.

#### 5.1.1 Comparison of sensitivity and specificity at a 65% threshold

For PTH at 0–20 min, fitting model (1) in a Bayesian framework gives a summary sensitivity of 0.82 (95% CI: 0.64 to 0.94) and a summary specificity of 0.79 (0.67 to 0.89). The probability that sensitivity will be greater than 0.8 in a new population is 0.60, and for specificity, it is 0.44. The probability that both will be above 0.8 in a new population is just 0.20. In contrast, recall that for PTH measured at 1–2 h, there was a probability of 0.57 that both would be above 0.8 in a new population, because of the larger summary sensitivity (0.93) and specificity (0.81). Thus, the discrimination of the test is substantially better when measured at 1–2 h.

#### 5.1.2 Comparison of the calibration of post‐test probabilities at a 65% threshold

At 0–20 min post‐surgery, the internal–external cross‐validation approach suggests that NPV is consistently predicted to be above 0.90, whereas PPV is much lower (Table 7). Higher NPV and PPVs are generally predicted at 1–2 h (Table 7), because of the larger sensitivity and specificity, and thus are clearly preferred if they calibrate well in new populations.

When using option B to derive post‐test predictions, at 0–20 min the summary O/E from meta‐analysis model (16) is 0.96, which suggests the test slightly over‐predicts hypocalcaemia events, and the 95% prediction interval is wide (0.61 to 1.50). In contrast, at 1–2 h post‐surgery, the summary O/E was better at 1.01, and the 95% prediction interval was slightly narrower (0.68 to 1.50), although still wide.

Thus, in terms of both discrimination and calibration performances in new populations, the PTH test measured at 1–2 h appears preferable based on the evidence available.

### 5.2 Predictions involving additional study‐level and patient‐level covariates

We suggested options A to C as possible strategies to derive post‐test predictions in new populations, based on the existing meta‐analysis results. If internal–external cross‐validation indicates these are all unsuitable, for example, because of poor calibration of predictions in new populations, then further strategies are needed to tailor predictions more accurately. For example, additional study‐level covariates could be included alongside prevalence in bivariate meta‐regression model (11), to reduce heterogeneity further. Or alternatively, study‐level covariates (including prevalence) could be included in a bivariate meta‐regression extension of model (1), to explain heterogeneity in sensitivity and specificity, and thereby allow sensitivity and specificity to be predicted in a new population conditional on particular covariate values. For instance, accounting for the country of the study or the timing of the test may be included. If individual participant data (IPD) are available, one could also develop and validate a multivariable prediction model that contains the test value as a patient‐level covariate, alongside multiple patient‐level and study‐level variables. The use of meta‐analysis to examine the performance of multivariable prediction models is increasingly of interest 34, 35.

## 6 Discussion

Where heterogeneity exists, summary meta‐analysis results might not be fit for purpose as test accuracy in particular populations may differ considerably from the average performance. Meta‐analysts therefore need to be clearer about if and how their test accuracy results can be implemented in clinical practice. In this article, we have proposed approaches to improve the reporting and translation of meta‐analysis results for use in clinical practice. We have brought together a number of existing concepts. In particular, we suggest that meta‐analysts should derive and report prediction intervals for the *c* statistic, sensitivity, specificity and O/E in new populations. Probability statements should also be made about the likelihood of acceptable test performance in new populations. Further, by omitting one study during each cycle of estimation, we suggest using internal–external cross‐validation to allow calibration of post‐test probabilities (PPV and NPV) to be examined in independent data on multiple occasions. This enables different strategies for calculating PPV and NPV to be formally evaluated and compared, to identify the best approach for deriving them in clinical practice.

Of course, the true impact of a diagnostic or prognostic test is ultimately measured on how it improves patient outcomes 36. This is perhaps best evaluated through a randomised trial, where one group has access to test results and the other does not. Our framework can help prioritise which tests are worth investment of a trial, by revealing which tests perform consistently well in new populations in terms of discrimination and calibration of post‐test probabilities.

### 6.1 Limitations of our work

The use of prediction intervals to summarise calibration and discrimination performance will be more informative when most studies have a reasonable sample size, and there are a reasonable number of studies available. When there are fewer than about 10 studies, predictive distributions and regions might be wide, as seen in the overall calibration results for the PTH test (Table 5). With small numbers of studies, the between‐study variance is estimated with large uncertainty, and this will be propagated in subsequent prediction intervals and distributions. Frequentist and Bayesian inferences may also be less similar with few studies (see, e.g. PTH results in Table 6), as prior distributions become more informative.

Even with large numbers of studies, wide prediction intervals will arise when there is large between‐study heterogeneity in test performance. This indicates the test may be useful in some populations but not in others, and so identifying those populations in which it works well becomes important (Section 5.2). However, identifying the causes of heterogeneity is also problematic, as meta‐regression often has low power and is prone to ecological bias 37, 38.

Underpinning all our methods is the assumption that studies included in the meta‐analysis are from relevant populations for which the test is desired and that the reference standard for true disease (or outcome) status is suitable in all studies. The broader the inclusion criteria (e.g. different patient pathways, different countries and different reference standards), the more likely heterogeneity in test performance will be identified. Between‐study heterogeneity may also arise because of different methods of test measurement, implicit or explicit differences in the choice of threshold and the quality (risk of bias) of the studies 39. This is clearly unwanted. Meta‐analysis results are more applicable when studies are of good quality and are consistent in how the test is implemented. Indeed, prediction intervals are really only relevant when based on studies with low risk of bias, as otherwise, they are widened to account for additional heterogeneity due to bias. Meta‐analysts might therefore use a strict study inclusion criteria before applying the methods suggested in this paper. For brevity, we have not examined risk of bias for the examples in this paper, but future work may revisit this for these data.

A key proposal is to use internal–external cross‐validation to examine the calibration of predicted PPV and NPV in new populations. For options B and C, these predicted values are derived in the external validation study with use of the known (expected) prevalence in that population. However, when implementing options B and C in both our applied examples, we did not know the known (expected) prevalence, and so used the observed prevalences. As we replaced the known prevalence with the observed prevalence, one could argue that the findings for options B and C in our applied examples may still be optimistic of true performance. However, given that the observed prevalence in a study should (for cohort studies) be an unbiased estimate of the true prevalence, we think this is perhaps a minor issue, although further research of this issue is appealing.

### 6.2 Related and further work

We believe that, if they are proposed for use in clinical practice, meta‐analysis results about PPV and NPV (or meta‐analysis equations to derive PPV and NPV in new populations) should be externally validated in new data. In other words, external validation is needed to *independently* check the reliability of post‐test probabilities in target populations. This is currently not well discussed in the test accuracy meta‐analysis literature. In contrast, in the prediction model field, the need for external validation is more widely recognised, as calibration and discrimination of multivariable model predictions are prone to over‐optimism and may vary across populations. Indeed, in prediction model research, others have already considered meta‐analysis of discrimination and calibration statistics from multiple external validation studies 24, 34, 35.

For test research, given data from multiple studies, we therefore proposed the internal–external cross‐validation approach to evaluate calibration in external data, to avoid over‐optimism 26. This allows previously recommended strategies to derive PPV and NPV (e.g. based on meta‐analysis models proposed by Leeflang *et al.* 5 and Chu *et al.* 30) to be formally evaluated in independent data from new populations. We note that if one study is much larger than other studies, it is perhaps wise to always retain it for the meta‐analysis (and thus never exclude it for external validation). In this manner, each cycle of internal–external cross‐validation will retain the majority of the available data for obtaining the meta‐analysis equation to predict PPV and NPV, and so the equation is likely to be very similar in each cycle. In related work, Willis and Hyde 10, 40 also suggest that test accuracy meta‐analysis results should be tailored to new populations. They consider a probabilistic approach to study selection using information from the setting of interest; as such, only studies which are plausible for the setting are considered for meta‐analysis. The performance of this approach could also be evaluated using the internal–external cross‐validation framework.

An interesting issue is how to define ‘good’ discrimination and calibration performance. In our application, we considered the probability that a test's calibration (O/E) is between 0.9 and 1.1, and the probability that a test's discrimination *c* statistic is above 0.7 or 0.8 in new populations. Such criteria are subjective, but probability statements such as these allow multiple tests to be compared and thereby help identify the best test for use. Such test comparisons are best considered in the subset of studies that evaluate all the tests of interest, to ensure direct comparisons in the same sets of patients and populations 41.

In small samples, the normal sampling distribution for *c*_*i*_ in model (4) may be a poor approximation, especially if *c*_*i*_ estimates are close to 1. Other transformations may thus be considered in further work, such as logit(*c*_*i*_), although van Klaveren *et al.* 24 conclude that the original *c* statistic scale is preferred. Also, we note that the prediction intervals assume the true effects follow a (bivariate) normal distribution across studies. The validity of this assumption requires empirical evaluation, and other more flexible distributions may be required 42. Finally, in our PTH example, the binary outcome was hypocalcaemia by 48 h and was known for every patient. For other prognostic test situations, the outcome may be longer‐term and some patients may be censored before the time of interest. Thus, extension of our work to time‐to‐event data is required.

## 7 Conclusion

In conclusion, we have suggested approaches to improve the translation of test accuracy meta‐analysis results for clinical practice. Prediction intervals and probability statements are important ways to summarise a test's discrimination and calibration performance in new populations. Further, internal–external cross‐validation allows meta‐analysts to identify a reliable strategy for how clinicians should derive post‐test probabilities in their population.

## Supporting information

Supporting info item

## References

[1] Deeks JJ . Systematic reviews in health care: systematic reviews of evaluations of diagnostic and screening tests. BMJ 2001; 323:157–162. 11463691 10.1136/bmj.323.7305.157 PMC1120791

[2] Reitsma JB , Glas AS , Rutjes AW , Scholten RJ , Bossuyt PM , Zwinderman AH . Bivariate analysis of sensitivity and specificity produces informative summary measures in diagnostic reviews. Journal of Clinical Epidemiology 2005; 58:982–990. 16168343 10.1016/j.jclinepi.2005.02.022

[3] Harbord RM , Deeks JJ , Egger M , Whiting P , Sterne JA . A unification of models for meta‐analysis of diagnostic accuracy studies. Biostatistics 2007; 8:239–251. 16698768 10.1093/biostatistics/kxl004

[4] Chu H , Cole SR . Bivariate meta‐analysis of sensitivity and specificity with sparse data: a generalized linear mixed model approach. Journal of Clinical Epidemiology 2006;59 1331–1332. author reply 1332–1333. 17098577 10.1016/j.jclinepi.2006.06.011

[5] Leeflang MM , Deeks JJ , Rutjes AW , Reitsma JB , Bossuyt PM . Bivariate meta‐analysis of predictive values of diagnostic tests can be an alternative to bivariate meta‐analysis of sensitivity and specificity. Journal of Clinical Epidemiology 2012; 65:1088–1097. 22742916 10.1016/j.jclinepi.2012.03.006

[6] Harbord RM , Whiting P , Sterne JA , Egger M , Deeks JJ , Shang A , Bachmann LM . An empirical comparison of methods for meta‐analysis of diagnostic accuracy showed hierarchical models are necessary. Journal of Clinical Epidemiology 2008; 61:1095–1103. 19208372 10.1016/j.jclinepi.2007.09.013

[7] Noordzij JP , Lee SL , Bernet VJ , Payne RJ , Cohen SM , McLeod IK , Hier MP , Black MJ , PD Kerr , Richards ML , Lo CY , Raffaelli M , Bellantone R , Lombardi CP , Cohen JI , Dietrich MS . Early prediction of hypocalcemia after thyroidectomy using parathyroid hormone: an analysis of pooled individual patient data from nine observational studies. Journal of the American College of Surgeons 2007; 205:748–754. 18035257 10.1016/j.jamcollsurg.2007.06.298

[8] Higgins JP , Thompson SG , Spiegelhalter DJ . A re‐evaluation of random‐effects meta‐analysis. Journal of the Royal Statistical Society, Series A 2009; 172:137–159. 10.1111/j.1467-985X.2008.00552.x PMC2667312 19381330

[9] Riley RD , Higgins JP , Deeks JJ . Interpretation of random effects meta‐analyses. BMJ 2011; :d549. 21310794 10.1136/bmj.d549

[10] Willis BH , Hyde CJ . Estimating a test's accuracy using tailored meta‐analysis – how setting‐specific data may aid study selection. Journal of Clinical Epidemiology 2014; 67:538–546. 24447592 10.1016/j.jclinepi.2013.10.016

[11] Craig JV , Lancaster GA , Taylor S , Williamson PR , Smyth RL . Infrared ear thermometry compared with rectal thermometry in children: a systematic review. Lancet 2002; 360:603–609. 12241932 10.1016/S0140-6736(02)09783-0

[12] Dodd SR , Lancaster GA , Craig JV , Smyth RL , Williamson PR . In a systematic review, infrared ear thermometry for fever diagnosis in children finds poor sensitivity. Journal of Clinical Epidemiology 2006; 59:354–357. 16549256 10.1016/j.jclinepi.2005.10.004

[13] Hamza TH , van Houwelingen HC , Stijnen T . The binomial distribution of meta‐analysis was preferred to model within‐study variability. Journal of Clinical Epidemiology 2008; 61:41–51. 18083461 10.1016/j.jclinepi.2007.03.016

[14] Zeger SL , Liang KY , Albert PS . Models for longitudinal data: a generalized estimating equation approach. Biometrics 1988; 44:1049–1060. 3233245

[15] Pinheiro JC , Bates DM . Approximations to the log‐likelihood function in the nonlinear mixed‐effects model. Journal of Computational and Graphical Statistics 1995; 4:12–35.

[16] Statistical Software . Release 13.0. Stata Corporation. College Station, TX, 2013.

[17] Lunn DJ , Thomas A , Best N , Spiegelhalter D . WinBUGS – a Bayesian modelling framework: concepts, structure, and extensibility. Statistics and Computing 2000; 10:325–337.

[18] Lambert PC , Sutton AJ , Burton PR , Abrams KR , Jones DR . How vague is vague? A simulation study of the impact of the use of vague prior distributions in MCMC. Statistics in Medicine 2005; 24:2401–2428. 16015676 10.1002/sim.2112

[19] Browne WJ , Draper D . Implementation and performance issues in the Bayesian and likelihood fitting of multilevel models. Computational Statistics 2000; 15:391–420.

[20] Zwinderman AH , Bossuyt PM . We should not pool diagnostic likelihood ratios in systematic reviews. Statistics in Medicine 2008; 27:687–697. 17611957 10.1002/sim.2992

[21] Wei Y , Higgins JP . Bayesian multivariate meta‐analysis with multiple outcomes. Statistics in Medicine 2013; 32:2911–2934. 23386217 10.1002/sim.5745

[22] Bujkiewicz S , Thompson JR , Sutton AJ , Cooper NJ , Harrison MJ , Symmons DP , Abrams KR . Multivariate meta‐analysis of mixed outcomes: a Bayesian approach. Statistics in Medicine 2013; 32:3926–3943. 23630081 10.1002/sim.5831 PMC4015389

[23] Harrell FE . Regression Modeling Strategies, with Applications to Linear models, Logistic Regression, and Survival Analysis. Springer: New York, 2001.

[24] van Klaveren D , Steyerberg EW , Perel P , Vergouwe Y . Assessing discriminative ability of risk models in clustered data. BMC Med Res Methodol 2014; 14:5. 24423445 10.1186/1471-2288-14-5 PMC3897966

[25] DerSimonian R , Laird N . Meta‐analysis in clinical trials. Control Clin Trials 1986; 7:177–188. 3802833 10.1016/0197-2456(86)90046-2

[26] Steyerberg EW . Clinical Prediction Models: A Practical Approach to Development, Validation, and Updating, Springer: New York, 2009.

[27] Royston P , Parmar MKB , Sylvester R . Construction and validation of a prognostic model across several studies, with an application in superficial bladder cancer. Statistics in Medicine 2004; 23:907–926. 15027080 10.1002/sim.1691

[28] Debray TP , Moons KG , Ahmed I , Koffijberg H , Riley RD . A framework for developing, implementing, and evaluating clinical prediction models in an individual participant data meta‐analysis. Statistics in Medicine 2013; 32:3158–3158. 23307585 10.1002/sim.5732

[29] Hemingway H , Croft P , Perel P , Hayden JA , Abrams K , Timmis A , Briggs A , Udumyan R , KG Moons , Steyerberg EW , Roberts I , Schroter S , Altman DG , Riley RD . Prognosis research strategy (PROGRESS) 1: a framework for researching clinical outcomes. BMJ 2013; 346:e5595. 23386360 10.1136/bmj.e5595 PMC3565687

[30] Chu H , Nie L , Cole SR , Poole C . Meta‐analysis of diagnostic accuracy studies accounting for disease prevalence: alternative parameterizations and model selection. Statistics in Medicine 2009; 28:2384–2399. 19499551 10.1002/sim.3627

[31] Steyerberg EW , Moons KG , van der Windt DA , Hayden JA , Perel P , Schroter S , Riley RD , H Hemingway , Altman DG . Prognosis research strategy (PROGRESS) 3: prognostic model research. PLoS Med 2013; 10:e1001381. 23393430 10.1371/journal.pmed.1001381 PMC3564751

[32] Bleeker SE , Moll HA , Steyerberg EW , Donders ART , Derksen‐Lubsen G , Grobbee DE , KGM Moons . External validation is necessary in, prediction research: a clinical example. Journal of Clinical Epidemiology 2003; 56:826–832. 14505766 10.1016/s0895-4356(03)00207-5

[33] Selvin S 2008 Survival Analysis for Epidemiologic and Medical Research: A Practical Guide, Cambridge University Press: New York.

[34] Pennells L , Kaptoge S , White IR , Thompson SG , Wood AM . Assessing risk prediction models using individual participant data from multiple studies. American Journal of Epidemiology 2014; 179:621–632. 24366051 10.1093/aje/kwt298 PMC3927974

[35] Snell KIE , Hua H , Debray TP , Ensor J , Look MP , Moons KG , Riley RD . Multivariate meta‐analysis of individual participant data helps externally validate the performance and implementation of a prediction model. Journal of Clinical Epidemiology (2015). (submitted). 10.1016/j.jclinepi.2015.05.009 PMC4688112 26142114

[36] Ferrante di Ruffano L , Hyde CJ , McCaffery KJ , Bossuyt PM , Deeks JJ . Assessing the value of diagnostic tests: a framework for designing and evaluating trials. BMJ 2012; 344:e686. 22354600 10.1136/bmj.e686

[37] Schmid CH , Stark PC , Berlin JA , Landais P , Lau J . Meta‐regression detected associations between heterogeneous treatment effects and study‐level, but not patient‐level, factors. Journal of Clinical Epidemiology 2004; 57:683–697. 15358396 10.1016/j.jclinepi.2003.12.001

[38] Berlin JA , Santanna J , Schmid CH , Szczech LA , Feldman HI . Individual patient‐ versus group‐level data meta‐regressions for the investigation of treatment effect modifiers: ecological bias rears its ugly head. Statistics in Medicine 2002; 21:371–387. 11813224 10.1002/sim.1023

[39] Whiting PF , Rutjes AW , Westwood ME , Mallett S , Deeks JJ , Reitsma JB , Leeflang MM , Sterne JA , Bossuyt PM . QUADAS‐2: a revised tool for the quality assessment of diagnostic accuracy studies. Annals of Internal Medicine 2011; 155:529–536. 22007046 10.7326/0003-4819-155-8-201110180-00009

[40] Willis BH , Hyde CJ . What is the test's accuracy in my practice population? Tailored meta‐analysis provides a plausible estimate. Journal of Clinical Epidemiology 2014 DOI: 10.1016/j.jclinepi.2014.10.002. 10.1016/j.jclinepi.2014.10.002 25479685

[41] Takwoingi Y , Leeflang MM , Deeks JJ . Empirical evidence of the importance of comparative studies of diagnostic test accuracy. Annals of Internal Medicine 2013; 158:544–554. 23546566 10.7326/0003-4819-158-7-201304020-00006

[42] Lee KJ , Thompson SG . Flexible parametric models for random‐effects distributions. Statistics in Medicine 2008; 27:418–434. 17477434 10.1002/sim.2897

