# Supplementary material for: Summarising and validating test accuracy results across multiple studies for use in clinical practice
Source: Stat Med. 2015 Mar 20;34(13):2081–103. doi: 10.1002/sim.6471 (PMC4973708; doi:10.1002/sim.6471)
Supplement: Supplementary file 1 — Supporting info item [file SIM-34-2081-s001.docx]

**Supplementary material:**

**S1: Model (1) estimation**

Model (1) can be fitted in a frequentist framework using maximum likelihood estimation. This will produce, for example, $\hat{\beta}_{1}$ and $\hat{\beta}_{0}$ by marginalizing study-specific logit-sensitivity and logit-specificity over the random effects [[14](#_ENREF_14)]. This can be undertaken using, for example, PROC NLMIXED in SAS or the xtmelogit procedure in STATA (code available on request) [[4](#_ENREF_4), [15](#_ENREF_15), [16](#_ENREF_16)], which fit nonlinear mixed models by maximizing an approximation to the likelihood integrated over the random effects. Different integral approximations are available, with adaptive Gaussian quadrature our method of choice [[15](#_ENREF_15)]. This requires a number of quadrature points to be specified, with increasing estimation accuracy as the number of points increases, but at the expense of an increased computational time. We generally choose 10 quadrature points, and assume successful convergence of the optimization procedure when successive iteration estimates differed by < 10^-7^, resulting in parameter estimates and their approximate standard errors based on the second derivative matrix of the likelihood function.

Model (1) can alternatively be fitted in a Bayesian framework, for example using Markov Chain Monte Carlo estimation in WinBUGS [[17](#_ENREF_17)]. This additionally requires specification of prior distributions for the unknown parameters (i.e. $\beta_{1}$, $\beta_{0}$, and $\mathbf{\Omega}$ in model (1)) and thus allows external information to be incorporated. Vague prior distributions can also be specified, such as $N\left(0,100000\right)$ for $\beta_{1}$ and $\beta_{0}$; however, specifying a non-informative prior distribution for $\mathbf{\Omega}$ may be difficult [[18](#_ENREF_18), [19](#_ENREF_19)], as estimation of $\mathbf{\Omega}$ is dependent on the number of studies, which will often be small. Sensitivity analysis of the posterior estimates to the choice of prior distribution for $\mathbf{\Omega}$ is thus advised. Zwinderman et al. [[20](#_ENREF_20)] assume $\mathbf{\Omega}^{-1}\sim Wishart(\mathbf{S},t)$, a common conjugate prior distribution for precision matrices [[19](#_ENREF_19)]. The *t* represents the degrees of freedom, which essentially represents the prior number of studies, and given no prior information it is usually set to the smallest feasible value, which is 2 here. **S** is the scale matrix, which can be considered the prior mean estimate of $\mathbf{\Omega}$. In our Bayesian analyses of model (1), we used $\mathbf{\Omega}^{-1}\sim Wish\left(\left(\begin{matrix} 1 & 0 \\ 0 & 1 \end{matrix}\right),2\right)$ and then performed sensitivity analysis to the choice of this prior. Other specifications of the prior distribution for $\mathbf{\Omega}$ are also possible [[21](#_ENREF_21), [22](#_ENREF_22)].

**S2: Model (1) specification in WinBUGS, with application to the temperature data**

model{

for (i in 1:N){

logits[i,1:2] ~ dmnorm(mu[1:2],T[1:2,1:2])

sens[i] <- exp(logits[i,1])/(1+exp(logits[i,1]))

spec[i] <- exp(logits[i,2])/(1+exp(logits[i,2]))

tn[i] ~ dbin(spec[i],ncontrols[i])

tp[i] ~ dbin(sens[i],ncases[i])

}

pooledsens <- exp(mu[1])/(1+exp(mu[1]))

pooledspec <- exp(mu[2])/(1+exp(mu[2]))

tau[1:2,1:2] <- inverse(T[1:2,1:2])

tau2sens<- tau[1,1]

tau2spec<- tau[2,2]

cov <- tau[1,2]

corr <- cov/sqrt(tau2sens*tau2spec)

# priors

mu[1:2] ~ dmnorm(mn[1:2],prec[1:2,1:2])

T[1:2,1:2] ~ dwish(R[1:2,1:2],2)

# Predict new pair of sens and spec

logitsnew[1:2] ~ dmnorm(mu[1:2],T[1:2,1:2])

sensnew<-exp(logitsnew[1])/(1+exp(logitsnew[1]))

specnew<-exp(logitsnew[2])/(1+exp(logitsnew[2]))

probsensnew80<- 1-equals(max(sensnew, 0.8), 0.8)

probspecnew80<- 1-equals(max(specnew, 0.8), 0.8)

# to look at joint probability they will both be above 80%

minlogitssensspec<-min(logitsnew[1], logitsnew[2])

minsensspec <- exp(minlogitssensspec)/(1+exp(minlogitssensspec))

probbothnew80<-1 - equals(max(minsensspec, 0.8), 0.8)

}

# Data: tn=true negatives; tp=true positives.

list(N=11,mn=c(0,0),

prec=structure(.Data=c(0.001,0,0,0.001),.Dim=c(2,2)),

#R=structure(.Data=c(0.001,0,0,0.001),.Dim=c(2,2)),

R=structure(.Data=c(0.1,0,0,0.1),.Dim=c(2,2)),

ncontrols=c(167, 48, 12, 58, 24, 75, 453, 38, 195, 136, 20),

tn=c(155, 46, 12, 56, 24, 74, 445, 38, 193, 136, 20),

ncases=c(203, 18, 9, 42, 15, 103, 425, 27, 109, 87, 59),

tp=c(150, 9, 8, 30, 10, 53, 282, 7, 53, 48, 57))

# initial estimates

list(mu=c(0.7,0.08),

T=structure(.Data=c(0.31,0.28,0.28,0.47),.Dim=c(2,2)))

**S3:** Predictive (posterior) distributions for the true sensitivity and true specificity of the (a) ear temperature test and (b) PTH test measured 1-2 hours post-surgery, following Bayesian estimation of model (1)

| **(a) Ear temperature test for diagnosis of fever** |  |
| --- | --- |
|  |  |
|  |  |
| **(b) PTH test for onset of hypocalcaemia by 48 hours** | |
|  |  |

**S4:** Meta-analysis of *c* statistics for the PTH example (frequentist estimation of model (4)), with PTH measured 0-20 mins post-surgery


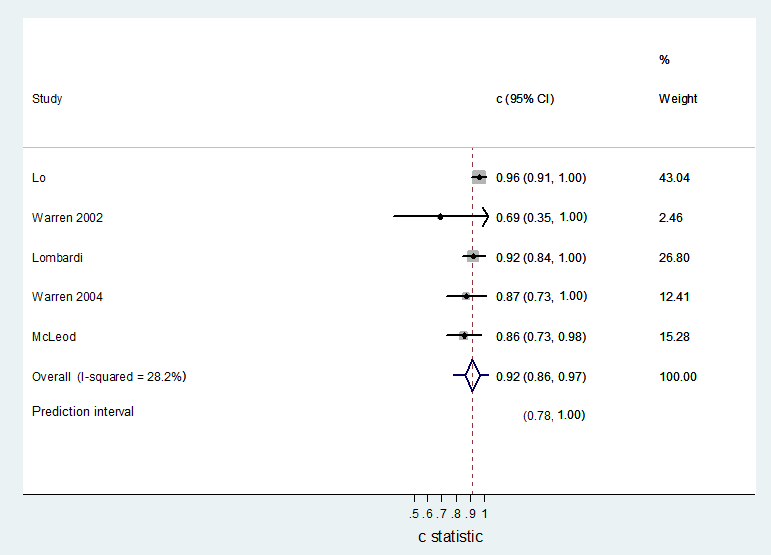


**S5: Model (17) specification in WinBUGS, with application to the temperature data**

model{

for (i in 1:N){

a[i] ~ dnorm(mu,T)

Prev[i]<- min(exp(a[i]+logit(p_E[i]))/(1+exp(a[i]+logit(p_E[i]))),0.99999)

d[i] ~ dbin(Prev[i] ,total[i])

}

T <- 1/(tau*tau)

# priors

tau ~ dunif(0,1)

mu ~ dnorm(0,0.00000001)

# Predict new true values when the predicted PPV is 0.97

anew ~ dnorm(mu,T)

predictlogitpnew<-anew + logit(0.97)

predictpnew <- exp(predictlogitpnew)/(1+exp(predictlogitpnew))

predictOE<- predictpnew/0.97

# calculate the probability that the predicted value gives an O/E of 0.9 to 1.1 #

probnew1point1<- 1 - equals(max(predictOE, 1.1), 1.1)

probnew0point9<- 1 - equals(max(predictOE, 0.9), 0.9)

probwithin09to11 <- probnew0point9 - probnew1point1

# calculated the probability that the true PPV is between 0.95 and 1

probnew0point95<- 1 - equals(max(predictpnew, 0.95), 0.95)

probnew1<- 1 - equals(max(predictpnew, 1), 1)

probwithin095to1 <- probnew0point95 - probnew1

}

}

# Data:

list(N=11,d=c(150, 9, 8, 53, 30, 10, 53, 48, 282, 7, 57 ),

total=c(162, 11, 8, 55, 32, 10, 54, 48, 290, 7, 57),

p_E = c(0.972233035,

0.974298419,

0.969536608,

0.971608299,

0.970804371,

0.973798816,

0.969229369,

0.968836728,

0.967579853,

0.963533847,

0.967232595

))

# initial estimates

list(mu=0.2, tau=0.2)

**S6: STATA code to fit model (1), and model (17)**

**Data set-up (temperature data) for model (1):**

| study | sens | tp | d | tn | nd | fp | fn | n | TRUE | spec |
| --- | --- | --- | --- | --- | --- | --- | --- | --- | --- | --- |
| 1 | 0 | 150 | 203 | 155 | 167 | 12 | 53 | 167 | 155 | 1 |
| 1 | 1 | 150 | 203 | 155 | 167 | 12 | 53 | 203 | 150 | 0 |
| 2 | 0 | 9 | 18 | 46 | 48 | 2 | 9 | 48 | 46 | 1 |
| 2 | 1 | 9 | 18 | 46 | 48 | 2 | 9 | 18 | 9 | 0 |
| 3 | 0 | 8 | 9 | 12 | 12 | 0 | 1 | 12 | 12 | 1 |
| 3 | 1 | 8 | 9 | 12 | 12 | 0 | 1 | 9 | 8 | 0 |
| 4 | 0 | 30 | 42 | 56 | 58 | 2 | 12 | 58 | 56 | 1 |
| 4 | 1 | 30 | 42 | 56 | 58 | 2 | 12 | 42 | 30 | 0 |
| 5 | 0 | 10 | 15 | 24 | 24 | 0 | 5 | 24 | 24 | 1 |
| 5 | 1 | 10 | 15 | 24 | 24 | 0 | 5 | 15 | 10 | 0 |
| 6 | 0 | 53 | 103 | 74 | 75 | 1 | 50 | 75 | 74 | 1 |
| 6 | 1 | 53 | 103 | 74 | 75 | 1 | 50 | 103 | 53 | 0 |
| 7 | 0 | 282 | 425 | 445 | 453 | 8 | 143 | 453 | 445 | 1 |
| 7 | 1 | 282 | 425 | 445 | 453 | 8 | 143 | 425 | 282 | 0 |
| 8 | 0 | 7 | 27 | 38 | 38 | 0 | 20 | 38 | 38 | 1 |
| 8 | 1 | 7 | 27 | 38 | 38 | 0 | 20 | 27 | 7 | 0 |
| 9 | 0 | 53 | 109 | 193 | 195 | 2 | 56 | 195 | 193 | 1 |
| 9 | 1 | 53 | 109 | 193 | 195 | 2 | 56 | 109 | 53 | 0 |
| 10 | 0 | 48 | 87 | 136 | 136 | 0 | 39 | 136 | 136 | 1 |
| 10 | 1 | 48 | 87 | 136 | 136 | 0 | 39 | 87 | 48 | 0 |
| 11 | 0 | 57 | 59 | 20 | 20 | 0 | 2 | 20 | 20 | 1 |
| 11 | 1 | 57 | 59 | 20 | 20 | 0 | 2 | 59 | 57 | 0 |

*** Model (1) ***

xtmelogit true sens spec, nocons || study: sens spec, nocons cov(un) binomial(n) refineopts(iterate(5)) intpoints(3)

**Data set-up (temperature data) for model (17):**

| study | npos | truepos | ppvpredict | logitppvpredict |
| --- | --- | --- | --- | --- |
| 1 | 162 | 150 | 0.981199 | 3.95486 |
| 2 | 11 | 9 | 0.939365 | 2.740335 |
| 3 | 8 | 8 | 0.965324 | 3.32641 |
| 4 | 55 | 53 | 0.957945 | 3.125804 |
| 5 | 32 | 30 | 0.964227 | 3.294132 |
| 6 | 10 | 10 | 0.964126 | 3.291197 |
| 7 | 54 | 53 | 0.980234 | 3.903842 |
| 8 | 48 | 48 | 0.958326 | 3.135309 |
| 9 | 290 | 282 | 0.971999 | 3.547114 |
| 10 | 7 | 7 | 0.955318 | 3.062474 |
| 11 | 57 | 57 | 0.99124 | 4.728709 |

*** Model (17) for PPV ***

xtmelogit truepos ,offset(logitppvpredict) || study: , binomial(npos)
